# Intestinal accumulation of microbiota-produced succinate caused by loss of microRNAs leads to diarrhea in weanling piglets

**DOI:** 10.1080/19490976.2022.2091369

**Published:** 2022-06-26

**Authors:** Xihong Zhou, Yonghui Liu, Xia Xiong, Jingqing Chen, Wenjie Tang, Liuqin He, Zhigang Zhang, Yulong Yin, Fengna Li

**Affiliations:** a Key Laboratory of Agro-ecological Processes in Subtropical Region, Institute of Subtropical Agriculture, the Chinese Academy of Sciences, Changsha, Hunan, China; b Hunan Provincial Key Laboratory of Animal Intestinal Function and Regulation, College of Life Sciences, Hunan Normal University, Changsha, Hunan, China; c Laboratory Animal Center of the Academy of Military Medical Sciences, Beijing, China; d Animal Breeding and Genetics Key Laboratory of Sichuan Province, Sichuan Animal Science Academy, Chengdu, Sichuan, China; e State Key Laboratory for Conservation and Utilization of Bio-Resources in Yunnan, School of Life Sciences, Yunnan University, Kunming, Yunnan, China

**Keywords:** Diarrhea, microbiome, microRNAs, piglets, succinate

## Abstract

Diarrheal disease is a common health problem with complex causality. Although diarrhea is accompanied by disturbances in microbial diversity, how gut microbes are involved in the occurrence of diarrhea remains largely unknown. Here, using a pig model of post-weaning stress-induced diarrhea, we aim to elucidate and enrich the mechanistic basis of diarrhea. We found significant alterations in fecal microbiome, their metabolites, and microRNAs levels in piglets with diarrhea. Specifically, loss of ssc-miRNA-425-5p and ssc-miRNA-423-3p, which inhibit the gene expression of fumarate reductase (*frd*) in *Prevotella* genus, caused succinate accumulation in piglets, which resulted in diarrhea. Single-cell RNA sequencing indicated impaired epithelial function and increased immune response in the colon of piglet with diarrhea. Notably, the accumulated succinate increased colonic fluid secretion by regulating transepithelial Cl-secretion in the epithelial cells. Meanwhile, succinate promoted colonic inflammatory responses by activating MyD88-dependent TLR4 signaling in the macrophages. Overall, our findings expand the mechanistic basis of diarrhea and suggest that colonic accumulation of microbiota-produced succinate caused by loss of miRNAs leads to diarrhea in weanling piglets.

## Introduction

Diarrheal diseases are a global health problem in humans and livestock. Although the mechanisms of diarrhea has been studied for decades, diarrhea still causes substantial mortality and morbidity in humans. This is because the causes of diarrhea are complex, including infection with bacteria and viruses, adverse effects of drugs, alternations of diet composition, and intestinal inflammatory and autoimmune conditions.^1^ Meanwhile, the diarrhea incidence was significantly increased after antibiotic use was gradually banned worldwide in animal husbandry. Diarrhea can be classified as acute or chronic diarrhea based on the duration, and classified as watery or fatty or inflammatory diarrhea based on the characteristics of the stools. Secretory diarrheas, occurred acutely and caused by reduced absorption or increased secretion of water from the digestive tract, are one of the most important subtypes of diarrhea, particularly in children.^1^ As pigs have metabolic and genetic features similar to those of humans, they are emerging as an attractive and accurate biomedical model for the study of diseases.^2,3^ Notably, post-weaning piglets with high incidence of secretory diarrhea could be an appropriate model for studying the mechanisms involved in diarrhea, since infant weaning is one of the major causes of diarrhea in children.^4,5^

Diarrheal diseases are accompanied by disturbances in gut microbial diversity.^6,7^ Patients with diarrhea had high abundance of *Enterobacteriaceae* and low levels of UniFrac distances based on the beta-diversity values.^8^ Diarrheal piglets showed reduced richness and diversity of microbiota and significant alterations of microbiota composition.^9^ Alterations in microbes further disturb colonic metabolism, resulting in adverse changes, such as carbohydrate over-accumulation and short-chain fatty acid deprivation, which may lead to diarrhea.^6^ Notably, weaning-induced stress in piglets could lead to post-weaning diarrhea and gut dysbiosis. Interestingly, fecal microbiota transplantation (FMT) from healthy piglets to weaned piglets decreases the incidence of diarrhea.^9^ These results confirm that the microbiota plays an important role in the development of diarrhea. However, the precise mechanisms underlying FMT and how the alterations of microbes in association with their metabolites involved in the development of diarrhea remain unclear.

Recently, numerous studies have found that the intestinal contents and feces contain abundant microRNAs (miRNAs).^10–13^ These miRNAs are critical for regulating microbial homeostasis. Importantly, we and others have found that the feces of diseased subjects or subjects who have recovered from disease might be enriched with miRNAs having therapeutic or preventive effects.^14,15^ These miRNAs exert beneficial effects in a microbiome-dependent manner, including affecting the growth of specific microbes and their metabolism. To explore whether and how fecal miRNAs and microbes might be involved in the regulation of stress-induced diarrhea in weaning piglets, we examined the gut microbiome composition and miRNA richness in weaned piglets with or without diarrhea in this study. We found alterations in the fecal microbiome and their metabolites in piglets with diarrhea. Importantly, we found that loss of ssc-miRNA-425-5p and ssc-miRNA-423-3p, which inhibit the gene expression of fumarate reductase (*frd*) in *Prevotella* genus, caused succinate accumulation in piglets, which resulted in diarrhea. Our findings enrich the mechanistic basis of diarrhea and suggest that miRNAs inhibit colonic accumulation of microbiota-produced succinate to suppress diarrhea in weanling piglets.

## Results

### Piglets with diarrhea showed impaired intestinal morphology and inflammation

**Figure 1.**
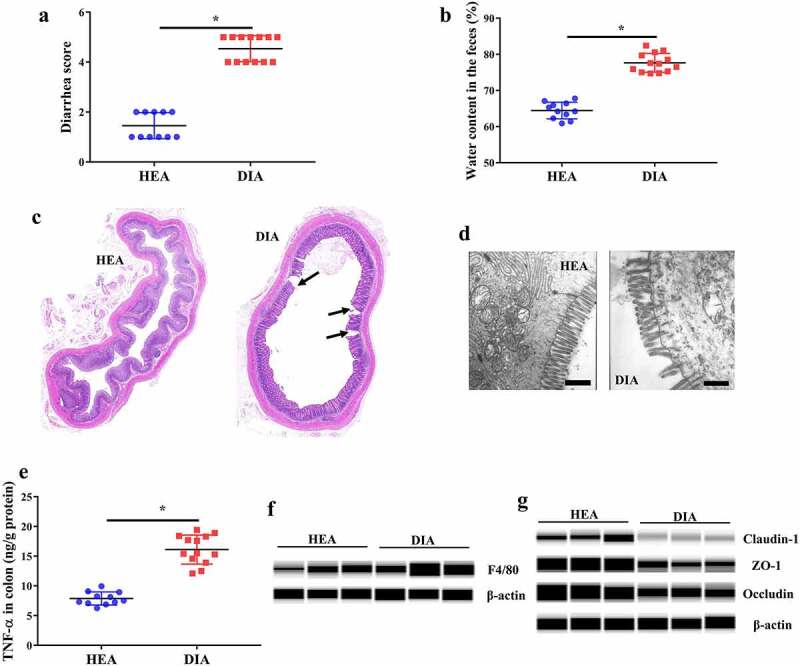
Piglets with diarrhea showed impaired intestinal morphology and inflammation. **a** Diarrhea score in weanling piglets. **b** Water content in the feces of weanling piglets. **c** Representative colonic morphology of the HE staining results. Arrow, loss of shape in colonic villi. **d** Representative colonic morphology under the transmission electron microscope. Arrow, unregularly aligned microvilli. Scale bar, 1 μm. **e** TNF-α level in colonic tissue. **f** Qualification of F4/80 protein expression. **g** Qualification of tight junction protein expression. HEA, healthy piglets without diarrhea (n = 11); DIA, diarrheal piglets (n = 13). Data are represented as mean ± SEM. *P < .05, determined by two-tailed Student’s *t*-test.

Piglets with diarrhea had higher diarrhea score and fecal water content than those in piglets without diarrhea (healthy piglets) (Figure 1aand b). To explore changes in the colonic tissue of piglets with diarrhea, we first observed the intestinal morphology. Hematoxylin–eosin (HE) staining results showed partial loss of shape (Figure 1c) in colonic villi and transmission electron microscopy exhibited irregularly aligned microvilli (Figure 1d) in piglets with diarrhea, suggesting that diarrheal piglets had impaired colonic histomorphology. We then explored whether this impaired morphology was associated with an inflammatory response. We found that the TNF-α concentration in colonic tissue was significantly increased in piglets with diarrhea (Figure 1e). Furthermore, protein qualification and immunohistochemical staining showed increased expression levels of F4/80 protein in piglets with diarrhea (Figure 1f, Supplementary Fig 1), indicating increased infiltration of macrophages in colonic tissue. We also found decreased expression of tight junction proteins (Claudin-1, ZO-1, and Occludin) in piglets with diarrhea, suggesting impaired epithelial function.

### Piglets with diarrhea exhibit alterations in the fecal microbiome and metabolites

**Figure 2.**
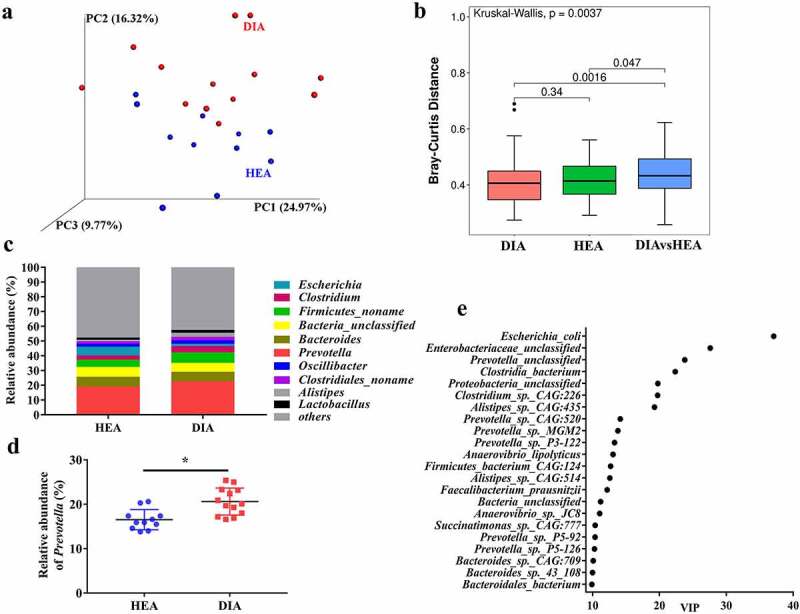
Piglets with diarrhea exhibit alterations in the fecal microbiome. **a** PCoA based on weighted UniFrac distance. **b** Beta-diversity (Bray-Curtis distance) at the gene level. **c** Relative abundance of bacteria classified at a genus-level taxonomy. **d** Relative abundance of *Prevotella* via fecal microbe quantification using qPCR, Data are represented as mean ± SEM. *P < .05, determined by two-tailed Student’s *t*-test. **e** VIP scores of PLS-DA (The top 20 species were presented). HEA, healthy piglets without diarrhea (n = 11); DIA, diarrheal piglets (n = 13).

To explore whether the intestinal microbiota in piglets with diarrhea was altered, we investigated the composition of the fecal microbiome using metagenomic sequencing. The results showed no significant difference in bacterial alpha diversity between diarrheal and healthy piglets (Supplementary Fig. 2a). Notably, principal coordinates analysis based on weighted UniFrac distance indicated that the overall bacterial structure in diarrheal piglets was separated from that in healthy piglets (Figure 2a). Moreover, the beta diversity of the microbiome in diarrheal piglets was significantly altered when compared with that of healthy piglets (Figure 2b). Furthermore, partial least squares discriminant analysis (PLS-DA) revealed a distinct clustering pattern between diarrheal and healthy piglets (Supplementary Fig. 2b).

To identify the changes in specific microbes, we compared the relative abundance of microbial species identified in the feces of diarrheal and healthy piglets. We found that relative enrichment in Bacteroidetes, a phylum comprising major succinate-producing species in the intestine,^16,17^ was increased in diarrheal piglets (Supplementary Fig. 2c). Notably, *Prevotella* was the most enriched genus, and its abundance was increased in piglets with diarrhea (Figure 2c). We further analyzed the enrichment of *Prevotella* via fecal microbe quantification using qPCR and confirmed their increase (Figure 2d). The variable importance in projection (VIP) score for the microbiota showed that *Escherichia coli* (*E. coli*) contributed significantly to group separation (Figure 2e). Surprisingly, *E. coli* enrichment was lower in diarrheal piglets than in healthy piglets (Supplementary Fig. 2d). Notably, the VIP score also exhibited that among the top 20 species contributing to group separation, six species belonged to the genus *Prevotella* (Figure 2e). Kyoto Encyclopedia of Genes and Genomes (KEGG) analysis indicated that alanine, aspartate, and glutamate metabolism was one of the key metabolic pathways affected by microbiota changes in diarrheal piglets (Supplementary Fig. 2e). Enterotoxigenic *E. coli* (ETEC)-induced diarrhea are the most prevalent in postweaning piglets.^18^ We found that expression of *LTb* gene encoding heat-labile toxin, and *STa* and *STb* genes encoding heat-stable toxins, as well as heat stable toxins content was undetectable in the feces and colonic content from both healthy and diarrheal piglets. These results suggested that the diarrhea was not ETEC-induced. To further explore whether levels of lipopolysaccharide (LPS), a product of *E. coli* and a common inducer of diarrhea,^19,20^ was increased in piglets with diarrhea, we determined its concentration in the feces and colonic content. However, the concentration of LPS did not differ between diarrheal and healthy piglets (Supplementary Fig.2 f, g), suggesting that the diarrhea was not LPS-induced.

**Figure 3.**
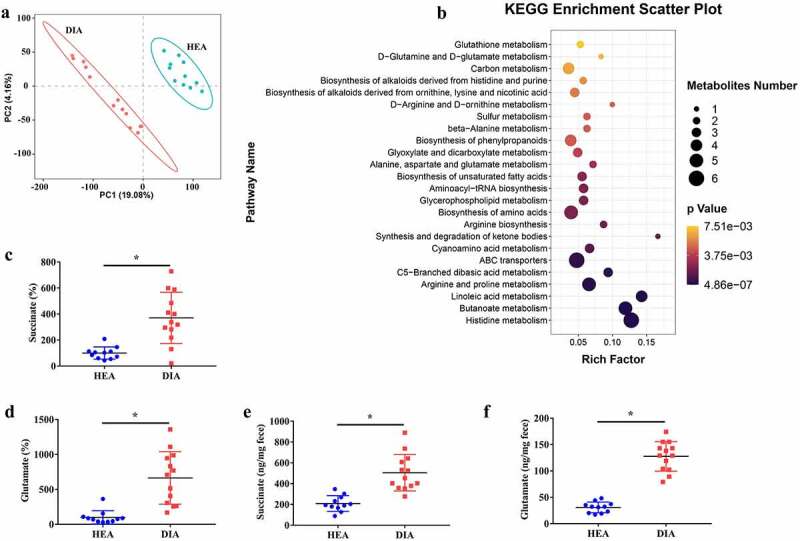
Piglets with diarrhea exhibit alterations in the fecal metabolites. **a** PLS-DA score plot. **b** KEGG enrichment scatter plot. **c-d** Relative levels of succinate (c) and glutamate (d) in the feces based on the untargeted metabolomics analysis. **e-f** Levels of succinate (e) and glutamate (f) in the feces determined by Colorimetric Assay Kit. Data are represented as mean ± SEM. *P < .05, determined by two-tailed Student’s *t*-test. HEA, healthy piglets without diarrhea (n = 11); DIA, diarrheal piglets (n = 13).

To identify the differences in metabolites between diarrheal and healthy piglets, an untargeted metabolomics approach was applied to fecal samples. The PLS-DA model exhibited significant separation of clusters between diarrheal and healthy piglets (Figure 3a). Analysis of differential metabolites showed dramatic alteration of metabolites in diarrheal piglets, with 1211 upregulated and 2426 downregulated metabolites (Supplementary Fig. 3). KEGG analysis revealed that most of the metabolites were enriched in energy and nutrient metabolism, such as histidine metabolism, butanoate metabolism, and arginine and proline metabolism (Figure 3b). Notably, alanine, aspartate, and glutamate metabolism was found to be affected based on both metagenomics and metabolomics analysis. Specifically, glutamate and succinate, two major intermediate metabolites involved in this key pathway, were significantly increased in diarrheal piglets (Figure 3cand d). Glutamate and succinate are also involved in most key pathways including butanoate metabolism, histidine metabolism, and arginine and proline metabolism. We further confirmed these changes through glutamate and succinate assays of fecal samples (Figure 3eand f). To explore whether the microbiota in diarrheal piglets prefer to produce succinate and glutamate, we performed *in vitro* fermentation experiment. We found that succinate and glutamate contents were significantly higher in the fermentation media of feces from diarrheal piglets than in those from healthy piglets (Supplementary Fig. 4a, b).

### Succinate accumulation induced diarrhea in piglets

**Figure 4.**
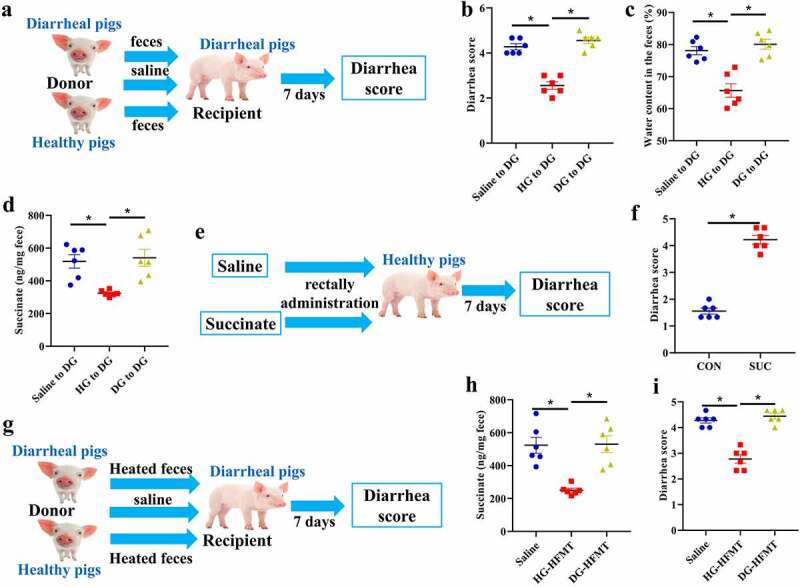
Effects of FMT and succinate on diarrhea in piglets. **a** Schematic design. **b-d** Diarrhea score (b), fecal water content (c) and succinate concentration (d) in diarrheal piglets transplanted with feces collected from healthy piglets or diarrheal piglets. n = 6, Data are represented as mean ± SEM. *P < .05, determined by one-way ANOVA. Saline to DG, diarrheal piglets were administrated with saline; HG to DG, feces were transplanted from healthy piglets to diarrheal piglets; DG to DG, feces were transplanted from diarrheal piglets to diarrheal piglets. **e** Schematic design. **f** Diarrhea score of healthy piglets treated with succinate through rectal administration. n = 6, Data are represented as mean ± SEM. *P < .05, determined by two-tailed Student’s *t*-test. **g** Schematic design. **h, i** Diarrhea score (h) and succinate concentration (i) in diarrheal piglets transplanted with heat-inactivated feces collected from healthy piglets or diarrheal piglets. n = 6, Data are represented as mean ± SEM. *P < .05, determined by one-way ANOVA. HG-HFMT, heat-inactivated feces were transplanted from healthy piglets to diarrheal piglets; DG-HFMT, heat-inactivated feces were transplanted from diarrheal piglets to diarrheal piglets.

To investigate whether the intestinal microbiome from diarrheal piglets had pathogenic properties, we transplanted feces from diarrheal piglets into healthy piglets and found that it did not induce diarrhea (Supplementary Fig. 5a-c). However, when we transplanted feces from healthy piglets into diarrheal piglets, diarrhea score, and water content in the feces were decreased (Figure 4a-c), suggesting that diarrhea was alleviated. Notably, the concentrations of succinate and glutamate were both decreased (Figure 4d, Supplementary Fig. 5d) in the feces of the originally diarrheal piglets. These results indicate that the metabolites succinate and glutamate may be associated with diarrhea occurrence. Thus, we investigated the effects of succinate or glutamate supplementation. Healthy piglets were fed diets supplemented with 1% succinate or glutamate. However, no diarrhea was observed after 7 days (Supplementary Fig. 5e, f). Previous studies suggest that most dietary succinate and glutamate are absorbed prior to the cecum;^21–23^ further, we found that the concentrations of succinate and glutamate were not changed in the feces after dietary supplementation (Supplementary Fig. 5e, f). Therefore, succinate or glutamate was administered through the rectum. As the succinate concentration in the intestinal lumen normally ranges from 1–3 mmol/kg,^24,25^ we treated the piglets with a much higher succinate concentration. We found that succinate could induce diarrhea (Figure 4e, f), whereas glutamate could not (Supplementary Fig. 5 g). These results indicate that succinate over-accumulation in the colon can induce diarrhea. Finally, we transplanted heat-inactivated feces from healthy piglets into diarrheal piglets, and found that it could alleviate diarrhea and decrease succinate concentration in the feces (Figure 4g-i). This finding indicated that other factors, such as miRNAs, may target the bacteria^14^ and affect their metabolism to produce succinate.

### Ssc-miRNA-425-5p and ssc-miRNA-423-3p inhibited the accumulation of succinate produced by Prevotella and prevented diarrhea

**Figure 5.**
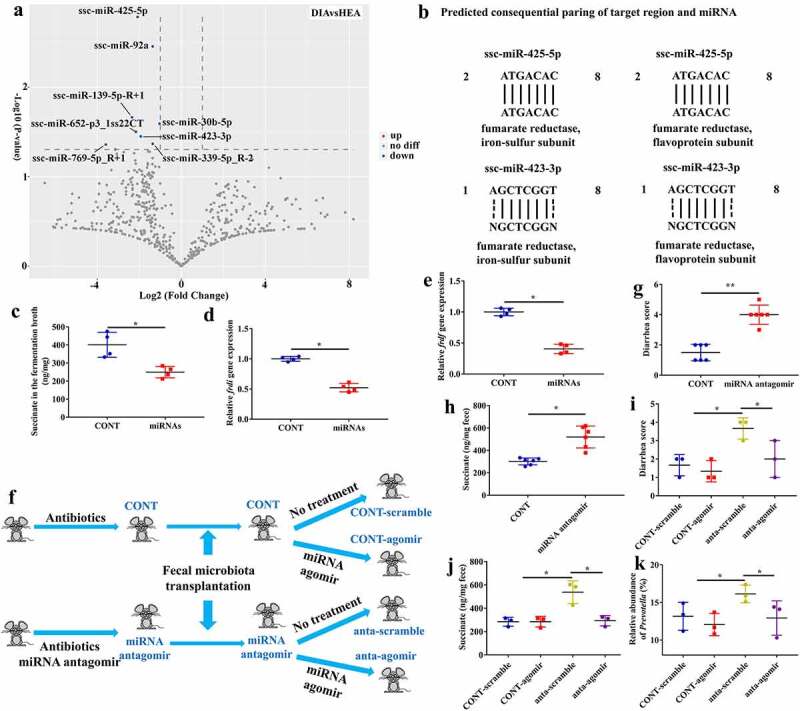
Ssc-miRNA-425-5p and ssc-miRNA-423-3p inhibited the accumulation of succinate produced by *Prevotella* and prevented diarrhea. **a** Differentially expressed miRNAs in the feces of healthy and diarrheal piglets. **b** Schematic diagram of the putative binding sites of the seed sequence of ssc-miRNA-425-5p and ssc-miRNA-423-3p in the two genes (*frdi* and *frdf*) of *Prevotella*. N = A, G, C or T. **c-e** Succinate content (c), gene expression of *frdi* (d) and *frdf* (e) in the fermentation broth of feces from diarrheal piglets after treated with ssc-miRNA-425-5p and ssc-miRNA-423-3p. n = 4, Data are represented as mean ± SEM. *P < .05, determined by two-tailed Student’s *t*-test. **f** Schematic design. **g, h** Diarrhea score (g) and succinate concentration (h) in mice pretreated with miRNA antagomirs and then transplanted feces from diarrheal piglets. n = 6, Data are represented as mean ± SEM. *P < .05, **P < .01, determined by two-tailed Student’s *t*-test. **i-k** Diarrhea score (i), succinate concentration (j) and *Prevotella* abundance (k) in mice further treated with miRNA agomirs. n = 3, Data are represented as mean ± SEM. *P < .05, determined by one-way ANOVA. CONT-scramble, control mice; CONT-agomir, mice treated with miRNA agomir; anta-scramble, mice pretreated with miRNA antagomirs and transplanted feces from diarrheal piglets; anta-agomir, mice pretreated with miRNA antagomirs and transplanted feces from diarrheal piglets, and then treated with miRNA agomir.

Previous studies have suggested that feces are abundant with extracellular vesicles containing small regulatory RNAs^26^ and miRNA profiles are altered when the intestine is under pathological conditions.^11,14,15^ Using transmission electron microscopy, we first confirmed that exosome-sized extracellular vesicles were present in the feces of both diarrheal and healthy piglets (Supplementary Fig. 6). To identify whether and which fecal miRNAs were affected during diarrhea, we performed small RNA sequencing. The results showed that a total of 731 miRNAs were present in the feces and that eight miRNAs were significantly downregulated in diarrheal piglets (Figure 5a). Previous studies have suggested that changes in miRNA profiles can affect the growth and metabolism of gut microbiota.^12,14^ Our results showed that succinate was increased in the feces of diarrheal piglets and that its over-accumulation in the colon could induce diarrhea. As succinate is one of the major metabolites of *Prevotella*, we investigated whether the significantly changed miRNAs could affect succinate metabolism in *Prevotella*. We performed a BLAST search for the seed sequence of the eight miRNAs against the sequences of the key enzyme involved in the synthesis of succinate in *Prevotella*, namely fumarate reductase [iron-sulfur subunit (*frdi*) and flavoprotein subunit (*frdf*)]. We found that among the eight miRNAs, ssc-miRNA-425-5p and ssc-miRNA-423-3p could target the *frd* genes (Figure 5b, Supplementary Fig. 7).

To investigate the effects of ssc-miRNA-425-5p and ssc-miRNA-423-3p on succinate production, we performed *an in vitro* fermentation experiment. Addition of these two miRNAs to the fermentation broth of the feces from diarrheal piglets significantly decreased the succinate content (Figure 5c) and the expression levels of *frdi* and *frdf* (Figure 5dand E). To further confirm the effects of these two miRNAs on succinate production and diarrhea, we first treated mice with miRNA-425-5p and miRNA-423-3p antagomir, along with antibiotics for 7 days. We then transplanted feces from diarrheal piglets into pre-treated mice (Figure 5f) and found that it induced diarrhea (Figure 5g) and increased the fecal succinate content (Figure 5h). Notably, when these mice were further treated with miRNA-425-5p and miRNA-423-3p agomir, diarrhea was alleviated (Figure 5i) and fecal succinate content was decreased (Figure 5j), accompanied by low abundance of *Prevotella* (Figure 5k).

### M^6^A methylation regulated the splicing process of pri-ssc-miRNA-425-5p and pri-ssc-miRNA-423-3p

Previous studies have demonstrated that m^6^A methylation could regulate miRNA expression by affecting primary miRNA processing.^27–29^ We first determined the expression of primary ssc-miRNA-425-5p (pri-ssc-miRNA-425-5p) and pri-ssc-miRNA-423-3p, as well as ssc-miRNA-425-5p and ssc-miRNA-423-3p in the colonic tissue. The results showed that expression of pri-ssc-miRNA-425-5p and pri-ssc-miRNA-423-3p were significantly increased, whereas expression of ssc-miRNA-425-5p and ssc-miRNA-423-3p were significantly decreased in diarrheal piglets compared with healthy piglets (Supplementary Fig. 8a, b). To further explore whether the decreased expression of ssc-miRNAs was affected by m^6^A methylation, we performed RNA immunoprecipitation using the m^6^A antibody. We found that both pri-ssc-miRNA-425-5p and pri-ssc-miRNA-423-3p bound to the m^6^A antibody were significantly decreased in the colonic tissue of diarrheal piglets (Supplementary Fig. 8c, d).

### Single-cell RNA sequencing indicated impaired epithelial function and elevated immune response in piglets with diarrhea

**Figure 6.**
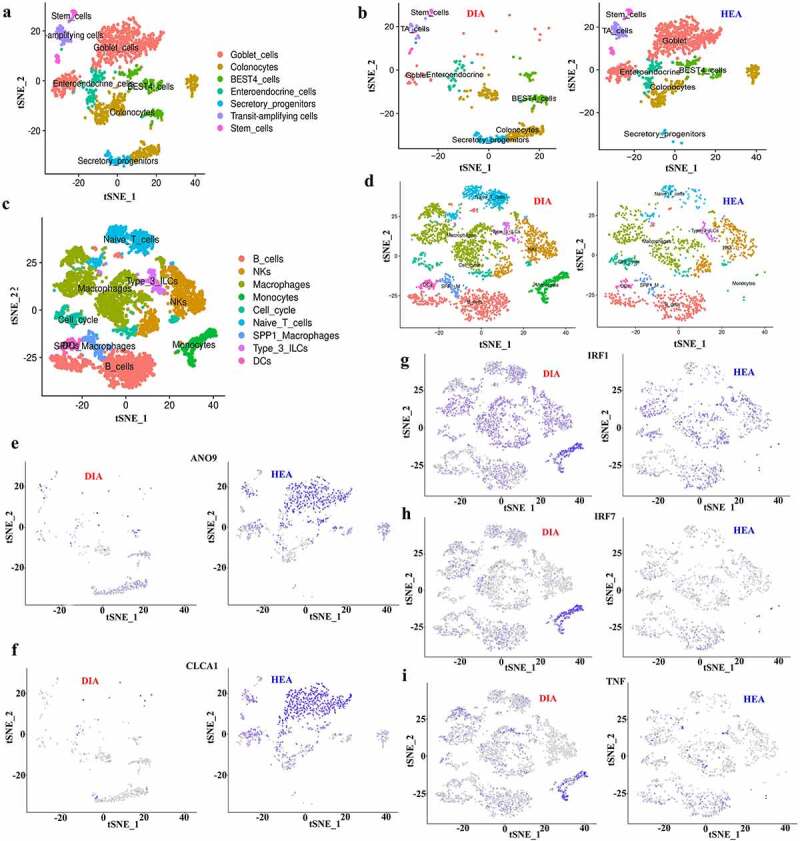
Single-cell RNA sequencing indicated impaired epithelial function and elevated immune response in piglets with diarrhea. **a,b** A t-SNE plot of epithelial cells of scRNA-seq data of colonic tissue from diarrheal piglet and healthy piglet showing clusters (**a**, merged results; **b**, separated results). **c, d** A t-SNE plot of immune cells of scRNA-seq data of colonic tissue from diarrheal piglet and healthy piglet showing clusters (**c**, merged results; **d**, separated results). **e-i** t-SNE plot showing expression of *ANO9* (e), *CLCA1* (f), *IRF1* (g), *IRF7* (h) and *TNF* (i) in colonic tissue from the indicated piglets at the single-cell level. HEA, healthy piglets without diarrhea; DIA, diarrheal piglets.

To characterize the baseline cellular diversity in the piglet colon and explore the effects of diarrhea, single-cell RNA sequencing (scRNA-seq) was performed using colonic tissue from piglet with diarrhea and healthy piglet. We sequenced 11,650 cells from piglets with diarrhea and 10,726 cells from healthy piglets. We then distinguished the cell types using unsupervised clustering. A combination of marker genes was used as previously described;^30^ the t‐SNE plots revealed eight putative major cell types, namely fibroblasts, immune cells, muscularis, neural/glial, epithelium, myofibroblasts, pericytes, and endothelium (Supplementary Fig.9a). The results showed that the relative cell number in each cell type, especially the epithelium and immune cells, was quite different between the groups (Supplementary Fig. 9b, c), indicating that the colons of diarrheal piglet exhibited distinct transcriptional signatures when compared with those of healthy piglet. Notably, diarrheal piglets had higher numbers of immune cells and fewer epithelial cells than those in the healthy piglets. The epithelial cells were further clustered into seven putative major subpopulations, namely goblet cells, colonocytes, BEST4 cells, enteroendocrine cells, secretory progenitors, transit-amplifying (TA) cells, and stem cells (Figure 6aand b), whereas the immune cells were clustered into monocytes, B cells, natural killer (NK) cells, macrophages, cycling cells, naïve T cells, SPP1 macrophages, type 3 innate lymphoid cells (ILCs), and dendritic cells (DCs) (Figure 6cand d). The statistics of GO enrichment revealed that most genes in epithelial cells were enriched in ion and proton transport (Supplementary Fig. 10a), whereas those in immune cells were enriched in defense response to bacteria and viruses, regulation of MyD88-dependent toll-like signaling pathway, and positive regulation of mononuclear cell migration (Supplementary Fig. 10b). Notably, we found that epithelial cell expression levels of *ANO9* and *CLCA1* genes, which are involved in Ca^2+^-activated Cl^−^ channels,^31^ were decreased in diarrheal piglets (Figure 6eand f). The expression levels of *IRF1, IRF7*, and *TNF* genes, which are downstream targets of the MyD88-dependent toll-like signaling pathway, were increased in diarrheal piglets (Figure 6gand i).

### Succinate increased fluid secretion in epithelium and immune response in macrophages

**Figure 7.**
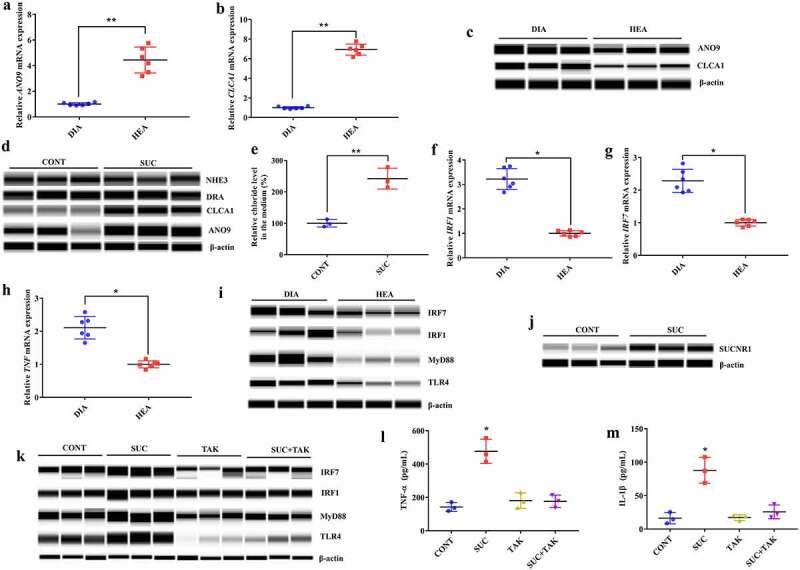
Succinate accumulation in the colon increased epithelial fluid secretion and the immune response in piglets with diarrhea. **a, b** Gene expression of *ANO9* (a) and *CLCA1* (b) in colonic tissue determined by RT-qPCR. n = 6, Data are represented as mean ± SEM. **P < .01, determined by two-tailed Student’s *t*-test. **c** Protein qualification of ANO9 and CLCA1 in colonic tissue by the Wes Simple Western System. **d** Protein qualification of ANO9, CLCA1, NHE3 and DRA in IPEC-J2 cells. **e** Relative chloride level in the culture medium. **f-h** Gene expression of *IRF1* (f), *IRF7* (g) and *TNF* (h) in colonic tissue determined by RT-qPCR. n = 6, Data are represented as mean ± SEM. *P < .05, **P < .01, determined by two-tailed Student’s *t*-test. HEA, healthy piglets without diarrhea; DIA, diarrheal piglets. **i** Protein qualification of IRF7, IRF1, MyD88 and TLR4 in colonic tissue. **j** Protein qualification of SUCNR1 in RAW264.7 cells. **k** Protein qualification of IRF7, IRF1, MyD88 and TLR4 in RAW264.7 cells. **l, m** Levels of TNF-α (l) and IL-1β (m) in the culture medium. n = 3, Data are represented as mean ± SEM. *P < .05, determined by one-way ANOVA. CONT, control cells; SUC, cells treated with disodium succinate; TAK, cells treated with TAK-242; SUC+TAK, cells treated with disodium succinate and TAK-242.

Succinate accumulation in the hindgut stimulates fluid secretion,^32^ which is driven by Cl-secretion through basolateral and apical Cl-channels and transporters.^1^ We first confirmed the decreased expression levels of *ANO9* and *CLCA1* genes using RT-qPCR in the colonic tissue of piglets with diarrhea (Figure 7aand b). However, further analysis of protein expression showed that ANO9 and CLAC1 levels were increased in diarrheal piglets (Figure 7c), contrary to their gene expression levels, indicating increased Cl^−^ secretion. To explore the effects of succinate on intestinal fluid secretion, we cultured IPEC-J2 cells and treated them with 20 mM disodium succinate. We found that the expression levels of ANO9 and CLAC1 proteins were significantly increased, whereas NHE3 and DRA expression levels did not change after treatment with disodium succinate (Figure 7d). We further found increased Cl^−^ concentration in the culture medium after disodium succinate treatment (Figure 7e). These results confirmed the effects of succinate on intestinal fluid secretion.

Succinate in the intestinal lumen is an important signal that triggers immune responses.^33^ We first confirmed the increased expression levels of *IRF1, IRF7*, and *TNF* genes in the colonic tissue of diarrheal piglets using RT-qPCR (Figure 7f–h). We further determined the protein expression levels of TLR4, MyD88, IRF1, and IRF7, and found that they were also significantly increased in diarrheal piglets (Figure 7i). As most *IRF1* and *IRF7* genes were expressed in monocytes and macrophages according to the scRNA-seq results, we cultured RAW264.7 cells and treated them with 50 mM disodium succinate to examine the effects of succinate on the MyD88-dependent toll-like signaling pathway. The results showed that protein expression levels of SUCNR1, TLR4, MyD88, IRF1, and IRF7 were significantly increased (Figure 7j, l) in RAW264.7 cells treated with disodium succinate. These results suggested that succinate might signal through SUCNR1 and act in synergy with TLR4.^34^ Meanwhile, TNF-α and IL-1β concentrations in the culture medium were significantly increased (Figure 7l, m). To further explore whether the MyD88-dependent TLR4 signaling pathway mediates the inflammatory response induced by succinate, we pretreated RAW264.7 cells with TAK-242 (resatorvid), a small molecule inhibitor of TLR4,^35^ and found that pretreatment with TAK-242 (1 μM) for 30 min diminished the effect of succinate on increasing the protein expression levels of MyD88, IRF1, and IRF7 (Figure 7k) and the production of TNF-α and IL-1β (Figure 7land m).

To explore whether miRNA-425-5p and miRNA-423-3p directly affected the expression of *ANO9, CLCA1, IRF1*, and *IRF7*, we first used TargetScan and found that these four genes were not potential targets of miRNA-425-5p and miRNA-423-3p. We then treated RAW264.7 and IPEC-J2 cells with miRNA-425-5p and miRNA-423-3p mimics, respectively. We found no changes in the expression levels of *ANO9* and *CLCA1* genes in IPEC-J2 cells, and in the expression levels of *IRF1* and *IRF7* genes in RAW264.7 cells (Supplementary Fig.11).

## Discussion

Diarrhea is estimated to occur in millions of patients, and diarrheal diseases cause losses of billions of dollars in healthcare expenditure every year globally.^36^ Various types of diarrhea are considered to be caused by different factors; therefore, elucidating the specific mechanisms and providing precise therapeutic methods for diarrhea is important. In this study, we used a model of post-weaning diarrhea in piglets and found that reduced expression levels of miRNAs in the feces were associated with significantly altered microbial composition and their metabolites. Notably, miRNA-425-5p and miRNA-423-3p, which were reduced in diarrheal piglets, showed modulating effects on *Prevotella* by directly targeting their specific genes and affecting the production of their metabolites, primarily succinate. Further, *in vivo* and *in vitro* experiments confirmed that over-accumulation of succinate in the colon could be a critical cause of diarrhea.

Gut microbiota dysbiosis is suggested to be an important reason for occurrence of diarrhea.^37^ Previous studies and our data showed that FMT decreased the diarrhea incidence in weaned piglets, confirming the beneficial effects of the microbiome on diarrhea.^9,37^ Interestingly, FMT with heat-inactivated feces from healthy piglets could still decrease the incidence of diarrhea in weaned piglets. However, FMT from diarrheal to healthy piglets did not induce diarrhea. As previous studies have demonstrated that colonic and fecal miRNAs could precisely target specific microbes,^13,15^ our results were reasonable based on this effect of miRNAs. In healthy piglets, the existence of ssc-miRNA-425-5p and ssc-miRNA-423-3p maintained the homeostasis of succinate production by *Prevotella* by regulating the gene expression of *frdi* and *frdf*. However, loss of these two miRNAs resulted in increased expression levels of *frdi* and *frdf* and an overproduction of succinate, which further resulted in diarrhea. When transplanting feces from healthy piglets into diarrheal piglets, ssc-miRNA-425-5p and ssc-miRNA-423-3p reach the colon, target *Prevotella*, and inhibit succinate production. However, upon transplanting feces from diarrheal piglets into healthy piglets, succinate production from *Prevotella* was inhibited by these two miRNAs in healthy piglets and succinate from the feces of diarrheal piglets was absorbed prior to the cecum and could not reach the colon. Importantly, after antagomirs were used to inhibit the expression of miRNA-425-5p and miRNA-423-3p in mice, FMT from diarrheal piglets to these mice resulted in over-accumulation of succinate and induced diarrhea. These results further confirmed the regulatory effects of miRNAs on succinate production in the colon. Notably, previous studies have indicated that miRNAs can promote the growth of certain microbes and improve the production of their metabolites.^12–15,38^ Here, we enriched the regulatory mechanisms and mode of action of miRNAs on microbes, as we found that miRNAs could also exert suppressive effects on microbial growth and metabolism. However, the detailed mechanisms by which miRNAs exert a suppressive or promoting effect on microbes require further studies.

Succinate can be produced by *Prevotella*, the most enriched genus in the colon.^21,39^ We found that this intermediate metabolite accumulated in the colon and that abundance of *Prevotella* was increased in diarrheal piglets. A previous study suggested that succinate accumulation in the hindgut stimulates water secretion and causes diarrhea.^32^ However, the major transporters or regulators involved in intestinal fluid secretion targeted by succinate have not been elucidated. Interestingly, scRNA-seq results indicated decreased expression levels of *CLCA1* and *ANO9* genes in epithelial cells, which encode proteins regulating transepithelial Cl^−^ secretion and driving intestinal fluid secretion;^1^ whereas further detection showed increased expression levels of the proteins encoded by these two genes. Therefore, our results suggest that succinate increases colonic fluid secretion by targeting CLCA1 and ANO9 at the protein level, whereas decreased expression level of these two genes is most likely a protective mechanism to inhibit excessive fluid secretion.

Succinate is emerging as an important metabolite with pro-inflammatory effects, and its accumulation within the gut lumen is commonly associated with intestinal dysbiosis and inflammation.^25,40^ Our scRNA-seq results revealed an increased number of immune cells in the colonic tissue, suggesting that succinate could activate immune responses. Among these responses, regulation of the MyD88-dependent TLR4 signaling was one of the major pathways affected by diarrhea. This result is consistent with previous findings, suggesting that succinate induced an inflammatory response by activating MyD88-dependent TLR4 signaling.^41^ We further confirmed the indispensable role of TLR4 signaling in mediating the pro-inflammatory effects of succinate in macrophages. Although increased succinate uptake into macrophages is known to enhance inflammation,^42^ the detailed interactions between succinate and TLR4 require further study.

In conclusion, we found significant changes in miRNA expression, microbiota composition, and metabolites in the feces of weanling piglets with diarrhea. We demonstrated that loss of miRNAs, which are originally involved in maintaining succinate production by targeting specific genes in *Prevotella*, resulted in succinate over-accumulation. Accumulated succinate increased colonic fluid secretion by regulating transepithelial Cl^−^ secretion in epithelial cells. Moreover, succinate promoted colonic inflammatory responses by activating MyD88-dependent TLR4 in macrophages. Overall, our findings expand the existing knowledge on the mechanisms of diarrhea and provide potential strategies for the prevention or treatment of diarrhea.

## Materials and methods

### Ethics statement

Animal procedures were approved by the Protocol Management and Review Committee of the Institute of Subtropical Agriculture, Chinese Academy of Sciences (ISA2020003).

### Experiments for the selection of healthy and diarrheal piglets

Sixty cross-bred male piglets (Duroc×Landrace×Yorkshire) weaned at the age of 21 days were maintained for one week and had free access to feed and water. During this week, all the piglets were fed diet according to the nutrient requirements of the NRC (2012) without adding any antibiotics, and they were scored the severity of diarrhea. Diarrhea index was scored as follows: 1 = hard feces; 2 = normal consistency, no scours; 3 = , soft partially formed, mild scours; 4 = loose, semi-liquid, moderate scours; 5 = watery feces; as previously did.^43^ Those piglets with a score of 4 or 5 for three continuous days were designated as diarrheal piglets, while those piglets with a score of 1 or 2 for three continuous days were designated as healthy piglets. Fresh fecal samples were collected during the experiment. Colonic samples were collected for morphology observation, determination of inflammatory cytokines and tight junction protein expression after healthy and diarrheal piglets were slaughtered.

### Histological analysis

Distal colons were fixed with 4% formaldehyde (Cat#F111941, Aladdin) and slices of 8-μm sections were performed with hematoxylin and eosin (HE) staining to observe morphology, as previously described.^44^ Distal colons were fixed with 2.5% glutaraldehyde (Cat#G105907, Aladdin) and ultrathin sections were stained as previously described.^45^ Then, a Zeiss 902 transmission electron microscope was used to observe microvilli.

### Measurement of colonic inflammation cytokine and lipopolysaccharide (LPS) levels

The level of TNF-α, IL-1β, and LPS were determined using commercially available kits (Cat#473, Cat#12680 and Cat#22108, Jiangsu Meimian industrial Co., Ltd).

### Immunohistochemistry staining of F4/80 protein

The distal colons were fixed with 4% formaldehyde (Cat#F111941, Aladdin), embedded in paraffin and cut into slices. After permeabilized, samples were blocked with 1% w/v bovine serum albumin (Cat#23210, Pierce) and incubated with F4/80 antibody (Cat#bsm-34028 M, Bioss). The samples were mounted with neutral balsam (Cat#N116470, Aladdin) and the results were observed.

### Metagenomic sequencing and analysis

Fecal DNA extraction was performed using the E.Z.N.A.® Stool DNA Kit (Cat#D4015-02, Omega, Norcross). DNA library was constructed by TruSeq Nano DNA LT Library Preparation Kit (Cat#FC-121-4001, Illumina). DNA was fragmented by dsDNA Fragmentase (Cat#M0348S, NEB) and added with an A-base for ligation. Then PCR were performed to amplify the ligated products. The metagenome was constructed via *de novo* assembling the quality-filtered reads. Metagenomic contigs (coding regions) were predicted by MetaGeneMark v3.26. Based on the taxonomic and functional annotation (GO and KEGG) of unigenes, the differential analysis was carried out by Fisher’s exact test.

### Quantification of fecal microbes by qPCR

Fecal DNA extraction was performed using the QIAamp Fast DNAStool Mini Kit (Cat#51604, QIAGEN). OTUs were quantified using Universal PCR Master Mix (Cat#4304437, Applied Biosystems). Primer sequences were presented in Table S1.

### Untargeted metabolomics

Metabolites in feces were extracted and then analyzed using an UPLC system (SCIEX, UK). Metabolites were determined using a high-resolution tandem mass spectrometer TripleTOF5600plus (SCIEX, UK). The metabolites were annotated by matching the exact molecular mass data (m/z) based on the online KEGG, HMDB database. The metabolite identification was further validated using an in-house fragment spectrum library. Different metabolites were determined with student t-tests. Multiple tests using an FDR were performed and the P value were used for adjustment. The different variables were discriminated via conducting supervised PLS-DA.

### Assay of succinate and glutamate contents

Succinate and glutamate assay of colonic content and feces from piglets and mice were performed using Succinate and Glutamate Colorimetric Assay Kits (Cat#MAK184 and Cat#MAK330, Merck).

### In Vitro fermentation experiments

Fresh fecal samples were obtained from healthy and diarrheal piglets and transferred into an anaerobic chamber. Feces were diluted 1:10 (wt/vol) in anaerobic diluent, and then filtered through four layers of cheesecloth. Fecal inocula (1%) was mixed with the culture medium as previous described.^46^ To determine the effects of miRNAs on the production of succinate, ssc-miRNA-425-5p and ssc-miRNA-423-3p mimics were added into the mixture at a final concentration of 2 μM. The sequence of miRNA mimics was presented in Table S2. A corn-soybean basal diet for piglets was used as a substrate for fermentation. The mixtures were incubated at 37°C and supernatant samples were collected at 6 h of fermentation and were immediately frozen at −80°C until further analysis.

### Fecal microbiota transplantation (FMT) experiments

Crossbred piglets weaned at the age of 21 days were used and fresh feces were collected from diarrheal piglets with a score of 4 or 5 and healthy piglets with a score of 1 or 2 for three continuous days. The fecal suspension was prepared and FMT was performed as previously described.^37^ Briefly, fresh and heat-inactivated feces were homogenized in sterile saline and passed through four layers of cheesecloth. The collected slurry was added with sterile glycerol (Cat#G116208, Aladdin) at a final concentration of 10% and then stored in liquid nitrogen. In Experiment 1, 18 crossbred piglets weaned at the age of 21 days (with a diarrhea score of 1 or 2 from day 21 to 23) were randomly divided to three groups (six pens per group and one piglet per pen). The piglets received a sterile saline, or fecal suspension from healthy piglets, or fecal suspension from diarrheal piglets from the age of 24 to 28 days by oral administration every other day. In Experiment 2, 18 crossbred piglets weaned at the age of 21 days (with a diarrhea score of 4 or 5 from day 21 to 23) were randomly divided to three groups. The piglets received a sterile saline, or fecal suspension from healthy piglets, or fecal suspension from diarrheal piglets from the age of 24 to 28 days as Experiment 1 did. In Experiment 3, 18 crossbred piglets weaned at the age of 21 days (with a diarrhea score of 4 or 5 from day 21 to 23) were randomly divided to three groups. The piglets received a sterile saline, or heat-inactivated fecal suspension from healthy piglets, or heat-inactivated fecal suspension from diarrheal piglets from the age of 24 to 28 days as Experiment 1 did. The piglets received a volume of 2 mL fecal suspension with 10^8^ CFU/mL microbes. At the age of 31 days, diarrhea index was scored and fecal sample was collected for water content assay. In Experiment 4, a volume of 0.2 mL fecal suspension from diarrheal piglets were orally gavaged into recipient mice once daily for 7 days.

### Succinate or glutamate treatment experiments in piglets

Eighteen crossbred piglets weaned at the age of 21 days (with a diarrhea score of 1 or 2 from day 21 to 23) were randomly divided to three groups. The piglets were fed with a basal diet, a basal diet supplemented with either 1% succinate (Cat#C15873, Acros) or 1% glutamate (Cat#D304062, Acros) for 7 days. In addition, 18 crossbred piglets weaned at the age of 21 days (with a diarrhea score of 1 or 2 from day 21 to 23) were randomly divided to three groups. The piglets were rectally administrated with saline, succinate, or glutamate, respectively. In brief, 2 mg succinate or glutamate dissolved in 1.0 mL sterile saline was administrated via a soft rectal probe (6 Fr size) placed 50 mm into the rectum as previously did.^47^ Succinate or glutamate was administrated once a day for 7 continuous days and then diarrhea score was recorded for the following three days.

### Determination of exosomes in the feces

The exosome in the feces was purified as previously described.^12^ Briefly, feces from the piglets were diluted to 30 mg/mL with PBS, centrifuged at 12 000 *g* for 15 min and filtered through a 0.2-μm filter. The exosomes were observed with a Zeiss 902 transmission electron microscope.

### Small RNA sequencing

Fecal samples were used for total RNA extraction using a miRNA isolation kit with phenol (Cat#AM1561, Invitrogen). Multiplex Small RNA Library Prep Set (NEBNext) were used to construct the small RNA-seq libraries. PCR amplification were performed using LongAmp Taq 2× Master Mix (Cat#M0287L, NEB) and quality was determined on an Agilent Bioanalyzer 2100 system. Then, the library preparations were constructed on an Illumina Hiseq 2500 platform, All the 18 ~ 26 nucleotide-length unique sequences were mapped to specific species precursors in miRBase 22.0. Different miRNA expression was analyzed using Student t test.

### MiRNA target prediction

The seed sequence of significantly altered miRNAs expressed in the feces between diarrheal and healthy piglets were BLAST-searched against the sequence encoding *frd* in *Prevotella* for sequence pairing using the NCBI blast tool.

### Bacterial gene transcript quantification

Total bacterial RNA extraction was performed using TRIzol (Cat#10296010, Invitrogen) and then cDNA was synthesized using a cDNA Reverse Transcription Kit (Cat#4374966, Applied Biosystems). Gene transcript of *frd* was quantified using Universal PCR Master Mix (Cat#4304437, Applied Biosystems). Primer sequences were presented in Table S1.

### In vivo miRNA treatment

Six-week-old C57BL/6 J male mice were provided by SLAC Laboratory Animal Central (Changsha, China). Before experiments, all animals were acclimated under standard conditions for the first week and then used in the experiments. Mice were administrated intravenously by tail vein injection 50 mg/kg miRNA-425-5p and miRNA-423-3p agomir and antagomir (Sangon Biotech) every other day in 7-days experiment.

### Antibiotic treatment

The mice were orally gavaged with antibiotics (ampicillin 1 mg/mL, metronidazole 1 mg/mL, neomycin 1 mg/mL, vancomycin 0.5 mg/mL, and streptomycin 1 mg/mL) (Cat# A9518, Sigma-Aldrich; Cat#M3761, Supelco; Cat#V2002, Cat#N6386, Sigma-Aldrich; Cat#85886, Supelco) dissovled in 0.2 mL nuclease-free water for 7 consecutive days.

### Measurement of miRNA expression by qPCR

Total RNA from colonic tissue was isolated with Trizol (Cat#10296010, Invitrogen). MiRNA cDNA synthesis was performed by using a miRNA cDNA Synthesis kit (Cat#A28007, Applied Biosystems). Real-time PCR was performed by using Fast Master Mix and Advanced miRNA Assays (Cat#A25576, ThermoFisher). Primers used were listed in Table S3.

### Methylated RNA immunoprecipitation and MeRIP–quantitative polymerase chain reaction

Total RNA extracted from colonic tissue were purified by GenElute mRNA Miniprep Kit (Cat#MRN70, Sigma-Aldrich) and Methylated RNA Immunoprecipitation was performed with riboMeRIPTM m^6^A Transcriptome Profiling Kit (Cat#C11051-1, RiboBio). In brief, purified mRNA was firstly treated by RNA Fragmentation Buffer, and then mixed with RNase Inhibitor and IP buffer. The mixture was further incubated with anti-m^6^A antibody that was prebound to magnetic beads A/G. Finally, the m^6^A-antibody–bound RNA was collected after eluted by a mixture of IP buffer, RNase Inhibitor, N^6^-methyladenosine, and nuclease-free water. The m^6^A methylation level of pri-ssc-miRNA-425-5p and pri-ssc-miRNA-423-3p was quantified by real-time PCR after MeRIP.

### Single‐cell RNA sequencing

Colonic tissue was obtained from diarrheal and healthy piglets. The tissue was cut into 0.5 mm^2^ pieces and dissociated into single cells in a mixture solution of 2 mg/ml papain, 0.35% collagenase IV5, 120 Units/ml DNase I. After filtered through 30–70 μm stacked cell strainer, the cells were incubated with red blood cell lysis buffer and then resuspended in Dead Cell Removal Microbeads. Cell suspensions were loaded to 10x chromium by using 10X Genomics Chromium Single-Cell 3’ kit (V3) (Cat#1000269, 10X Genomics) for single-cell preparation and library construction as previously described.^48,49^ The library was multiplexed and sequenced on Illumina NovaSeq 6000 sequencing system.

Sequencing results were converted to FASTQ format using Illumina bcl2fastq software (version 2.20). The Cell Ranger pipeline was used for barcode processing and single-cell 3ʹgene counting. The Cell Ranger output was loaded into Seurat for scRNA-seq data clustering and analysis. The expression value of genes was calculated using the “Normalization” function of the Seurat software; Then, PCA analysis was performed and the top 10 PCs were used to perform clustering and t-SNE analysis; Clusters were further characterized by using selecting weighted Shared Nearest Neighbor (SNN) graph-based clustering method. Marker genes were identified with the Wilcoxon rank-sum test via the Find-All-Markers function in Seurat.

### Detailed characterization of intestinal scRNA-seq clusters

ScRNA-seq results were analyzed by unsupervised clustering to distinguish cell types. A combination of marker genes was used as previously described.^30^ Compartments included differential expression of immune (*PTPRC*), fibroblast (*THY1, COL1A2, VIM*), myofibroblast (*VIM, FOXF1, TAGLN*), neural/glial (*PHOX2B, HAND2, TUBB2B*), pericyte (*KCNJ8, ABCC9, TGS5*), endothelium (*PECAM1, CDH5, CLDN5*), epithelial (*EPCAM, FABP1*), and muscle (*MYH11, ACTG2*) genes. Further, epithelial cells were characterized as TA cells (*MKI67, UBE2C* and *TOP2A*), colonocytes (*FABP2, CEACAM1*, and *EPCAM*), enteroendocrine cells (*CHGA, TPH1*, and *NEUROD1*), goblet cells (*MUC2, SPDEF*, and *WFDC2*), stem cells (*LGR5, ASCL2*, and *SMOC2*), BEST4 cells (*CA7, CA4, BEST4*, and *OTOP2*) and secretory progenitor (*FOXA2*). Immune cells were characterized as monocytes (*CD14, FCN1*, and *CLEC12A*), macrophages (*MERTK, CTSC*, and *CTSD*), B cells (*CD19, CD79A*, and *IGH*), NK cells (*TRDC* and *IL2RB*), cycling cells (*MKI67* and *CENPF*), ILCs (*KLRB1, RORC*, and *ID2*) and DCs (*FLT*3 and *ETV6*).

### Determination of gene expression by quantitative real-time PCR

Total RNA extraction was performed using Trizol reagent and then cDNA was synthesized using the PrimeScript RT reagent kit (Cat#RR037A, Takara). RT-qPCR was performed using qPCR Master Mix (Cat#K0252, ThermoFisher), as previously described.^50^ The primer sequences used were showed in Table S4.

### Cell culture

RAW264.7 and IPEC-J2 cell lines were cultured in DMEM (Cat#11320033, Gibco) containing 10% FBS (Cat#10099, Gibco) and 1% penicillin/streptomycin (Cat#15140122, Gibco) in 5% CO_2_ at 37 ℃.

### In vitro succinate treatment

IPEC-J2 cell line was treated with 20 mM disodium succinate (Cat#S2378, Sigma-Aldrich) for 24 h and then samples were collected for the detection of protein expression. RAW264.7 cell line was treated with 50 mM disodium succinate for 24 h and/or TAK-242 (Cat#S7455, Selleck) for 30 min and then samples were collected for the detection of protein expression.

### Protein qualification using Wes simple western system

Total protein from the colonic tissue and cells were extracted and used for protein quantification using Wes Simple Western System (ProteinSimple) as previously described.^51^ Antibodies against ZO-1 (Cat#ab190085, Abcam), Claudin-1 (Cat#ab180158, Abcam), Occludin (Cat#ab216327, Abcam), F4/80 (Cat#bsm-34028 M, Bioss), ANO9 (Cat# ABV11856, Abcepta), CLAC1 (Cat#GTX66323, GeneTex), SUCNR1 (Cat#ab75105, Abcam), TLR4 (Cat#ab22048, Abcam), MyD88 (Cat#ab28763, Abcam), IRF1 (Cat#ab230652, Abcam), and IRF7 (Cat#bs-2994 R, Bioss) were used. The bands were detected using the “gel view” function of the Protein Simple software (ProteinSimple).

## Determination of chloride concentration

Chloride concentration in the cell culture medium was determined using commercially available kit (Cat#MAK023, Sigma-Aldrich).

### In vitro miRNA mimics treatment

RAW264.7 and IPEC-J2 cells were transiently transfected with 50 nM miRNA-425-5p and miRNA-423-3p mimics (Sangon Biotech) by using Lipofectamine 3000 (Cat#L3000015, ThermoFisher), respectively.

### Statistical analysis

Significance between treatments was analyzed using a two-tailed Student t test for two groups or one-way ANOVA followed by Student-Newman-Keuls post hoc test for multiple groups, using the data statistics SPSS Statistics 18.0 Software and GraphPad Prism 8.0 Software. All data are expressed as the mean ± SEM. P value < .05 was considered significant. No data were excluded in the final statistical analysis.

## Contributions

H.X.Z. designed the experiments; H.X.Z., H.Y.L., X.X., Q.J.C., J.W.T., and Q.L.H. performed the experiments; H.X.Z., G.Z.Z. and Q.L.H. analyzed the data. H.X.Z., Q.L.H. L.Y.Y. and N.F.L. wrote the manuscript with the help of all authors. All authors read and approved the final version of the manuscript.

## Supplementary Material

Supplemental Material

## Data Availability

The metagenomics sequence data have been deposited in the NCBI BioProject database (https://www.ncbi.nlm.nih.gov/bioproject/) under accession numbers PRJNA791695. Small RNA sequencing data have been deposited in the NCBI GEO database (https://www.ncbi.nlm.nih.gov/geo/) under accession number GSE192690. Single-cell RNA sequencing data have been deposited in the NCBI SRA database (https://www.ncbi.nlm.nih.gov/sra/) under accession numbers PRJNA792085. All other data is contained with the main manuscript and supplemental files.
