# Supplementary material for: Intestinal accumulation of microbiota-produced succinate caused by loss of microRNAs leads to diarrhea in weanling piglets
Source: Gut Microbes. 2022 Jun 26;14(1):2091369. doi: 10.1080/19490976.2022.2091369 (PMC9235893; doi:10.1080/19490976.2022.2091369)

**Supplementary Table 1 Primers for quantification of bacteria and bacterial gene transcript by qPCR**

| Gene | 5’-3’ Primer sequence |  |
| --- | --- | --- |
| *Prevotella 16S* | F: CACRGTAAACGATGGATGCC | |
|  | R: GGTCGGGTTGCAGACC | |
| *frdi* | F: ATGTAGCGCAGTCCATAGCC | |
|  | R: AAGGCAACGAGCCTTTCGTA | |
| *frdf* | F: GTAGTCTACCCAGATGCCGC | |
|  | R: GGCACAAAGAAGGGATCGGA | |
| *General bacteria* | F: CGGCAACGAGCGCAACCC | |
|  | R: CCATGTAGCACGTGTGTAGCC | |
| *Prevotella* | F: GGTTCTGAGAGGAAGGTCCCC | |
|  | R: TCCTGCACGCTACTTGGCTG | |

**Supplementary Table 2 Sequences of miRNA mimics, agomir and antagomir**

| Gene | 5’-3’ sequence |  |
| --- | --- | --- |
| mimics miRNA-425-5p | F: AAUGACACGAUCACUCCCGUUGA | |
|  | R: AACGGGAGUGAUCGUGUCAUUUU | |
| mimics miRNA-423-3p | F: AGCUCGGUCUGAGGCCCCUCAGU | |
|  | R: ACUGAGGGGCCUCAGACCGAGUU | |
| agomir-miRNA-425-5p | F: AAUGACACGAUCACUCCCGUUGA | |
|  | R: AACGGGAGUGAUCGUGUCAUUUU | |
| agomir-miRNA-423-3p | F: AGCUCGGUCUGAGGCCCCUCAGU | |
|  | R: ACUGAGGGGCCUCAGACCGAGUU | |
| antagomir-miRNA-425-5p | UCAACGGGAGUGAUCGUGUCAUU | |
| antagomir-miRNA-423-3p | ACUGAGGGGCCUCAGACCGAGCU | |

**Supplementary Table 3 Primers for quantification of miRNAs expression by qPCR**

| Gene | 5’-3’ Primer sequence |  |
| --- | --- | --- |
| ssc-miRNA-425-5p | F: AAGAGCGTAATGACACGATCAC | |
|  | R: CAGTGCAGGGTCCGAGGT | |
|  | RT:GTCGTATCCAGTGCAGGGTCCGAGGTATTCGCACTGGATACGACTCAACG | |
| ssc-miRNA-423-3p | F: TAATCGAAGCTCGGTCTGAGG | |
|  | R: CAGTGCAGGGTCCGAGGT | |
|  | RT:GTCGTATCCAGTGCAGGGTCCGAGGTATTCGCACTGGATACGACACTGAG | |
| U6 | F: GCTTCGGCAGCACATATACTAAAAT | |
|  | R: CGCTTCACGAATTTGCGTGTCAT | |
|  | RT: CGCTTCACGAATTTGCGTGTCAT | |

**Supplementary Table 4 Primers for quantification of gene expression by qPCR**

| Gene | 5’-3’ Primer sequence |  |
| --- | --- | --- |
| *ANO9* | F: GCTCATCAACTGCCCTGACT | |
|  | R: TTCAGGGCCACACACTTGTT | |
| *CLCA1* | F: TCTGCAGACAGTGGAGCAAG | |
|  | R: CAGTAAATGCGGAGCGAAGC | |
| *TNF* | F: GCCCTTCCACCAACGTTTTC | |
|  | R: CAAGGGCTCTTGATGGCAGA | |
| *IRF1* | F: CTTCGCTCCCACCGGGAATC | |
|  | R: ATCGGCCTGTGTGAATAGCC | |
| *IRF7* | F: CTTCGGAGACTGGCTTCTGG | |
|  | R: GCACAGCGGAAGTTGGTTTT | |
| *β-actin* | F: CTGCGGCATCCACGAAACT | |
|  | R: AGGGCCGTGATCTCCTTCTG | |

**Supplementary Fig.1 Representative results of immunohistochemistry staining with F4/80 protein in colonic tissue of piglets.** Yellow, F4/80 protein expression. Scale bar, 100 μm. HEA, healthy piglets without diarrhea; DIA, diarrheal piglets.


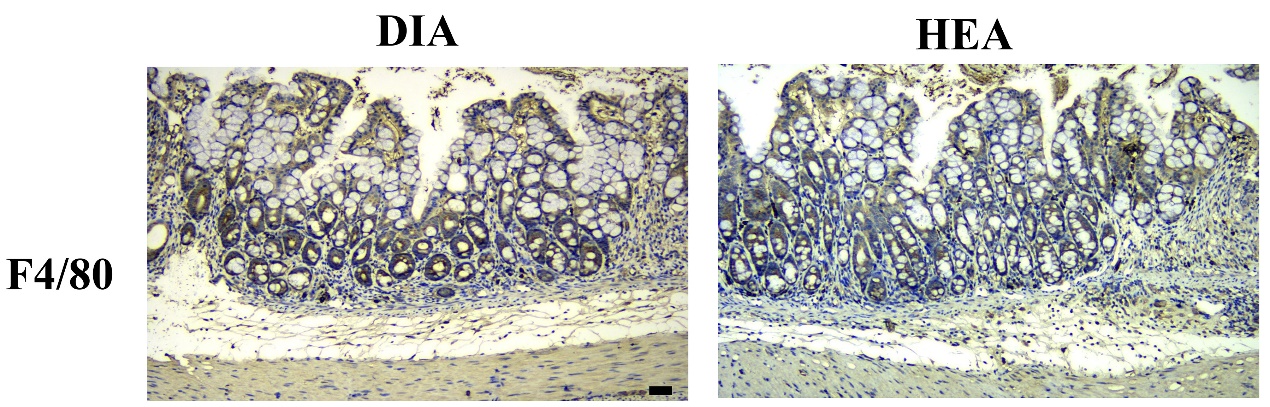


**Supplementary Fig.2 Piglets with diarrhea exhibit alterations in the fecal microbiome. a** Chao1 index. n=11 for healthy piglets and n=13 for diarrheal piglets. Data are represented as mean ± SEM and determined by two-tailed Student’s t-test. **b** PLS-DA analysis. **c** Relative abundance of bacteria classified at a phylum-level taxonomy. **d** Relative abundance of bacteria classified at a species-level taxonomy. **e** KEGG annotation of key altered metabolic pathways in diarrheal piglets. **f, g** LPS in colonic content (**f**) and feces (**g**). n=8. Data are represented as mean ± SEM and determined by two-tailed Student’s t-test. HEA, healthy piglets without diarrhea; DIA, diarrheal piglets.


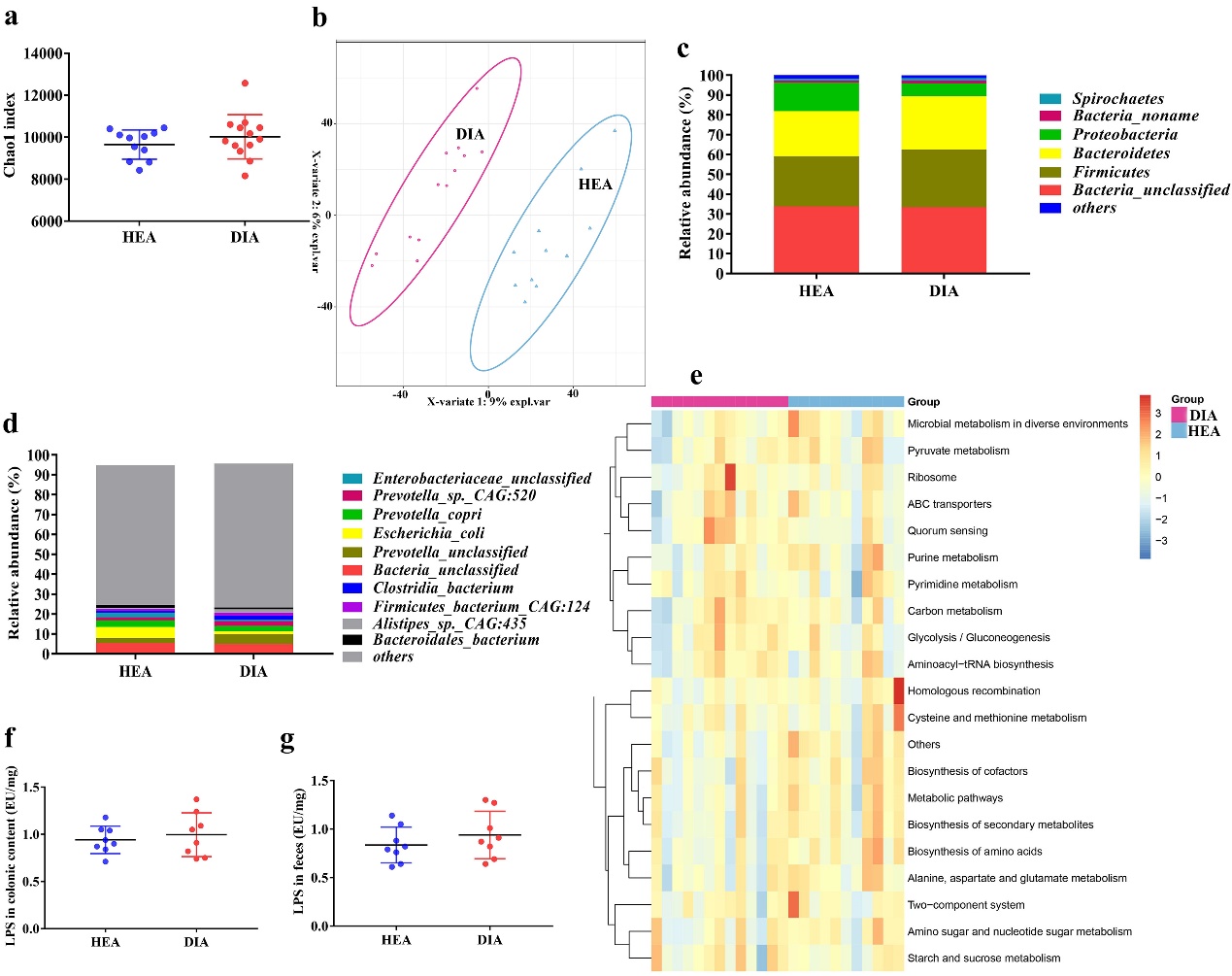


**Supplementary Fig.3 Altered metabolites in the feces of diarrheal piglets.**


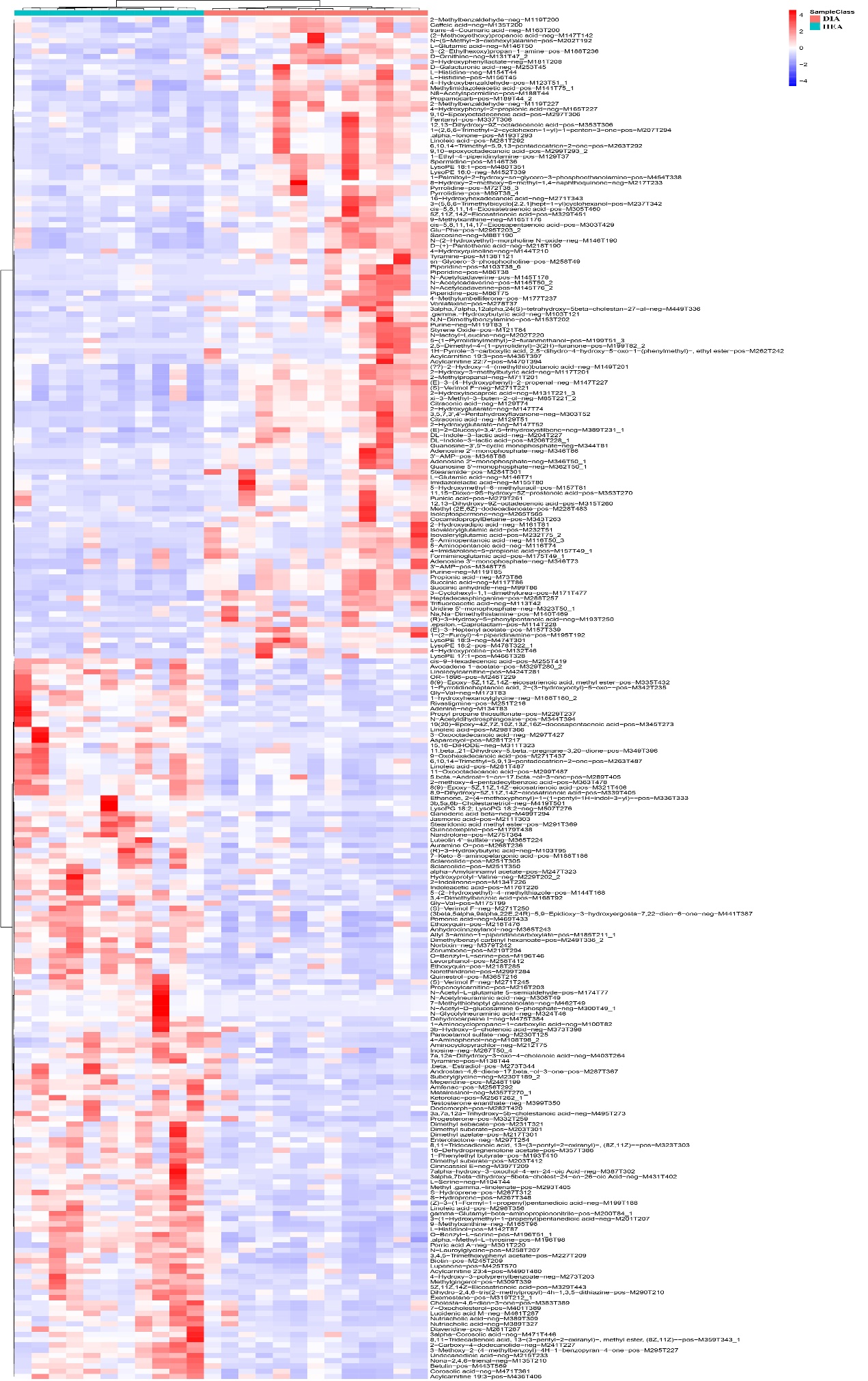


**Supplementary Fig.4 Succinate and glutamate contents in *in vitro* fermentation experiment.** Succinate (**a**) and glutamate (**b**) contents in the fermentation media of feces. n=4, Data are represented as mean ± SEM. **P*<0.05, determined by two-tailed Student’s t-test. HEA, feces from healthy piglets; DIA, feces from diarrheal piglets.


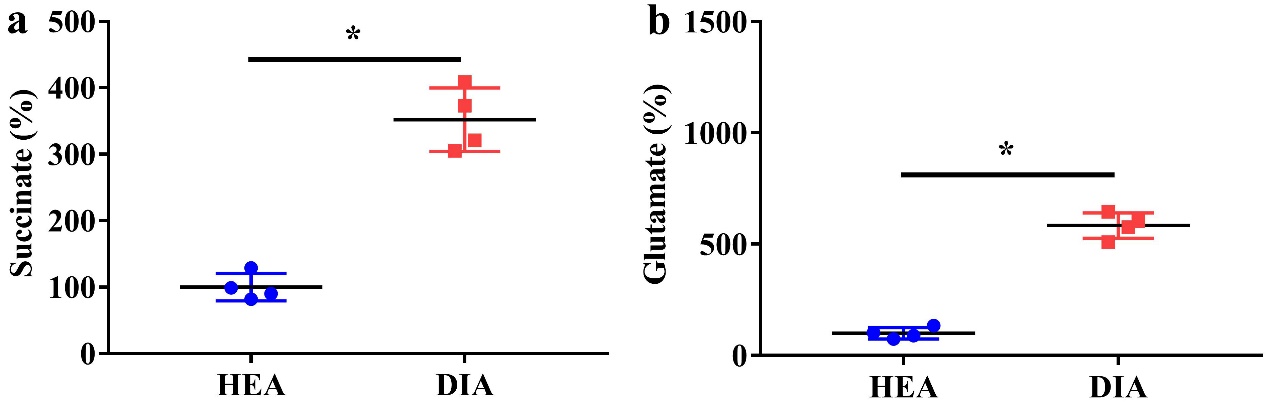


**Supplementary Fig.5** **Effects of FMT and succinate on diarrhea in piglets.** **a** Schematic design. **b, c** Diarrhea score (**b**) and fecal water content (**c**) in healthy piglets transplanted with feces collected from healthy piglets or diarrheal piglets. **d** Glutamate concentration in diarrheal piglets transplanted with feces collected from healthy piglets or diarrheal piglets. n=6, Data are represented as mean ± SEM. **P*<0.05, determined by one-way ANOVA. **e** Diarrhea score and colonic succinate concentration of healthy piglets treated with diets supplemented with succinate. **f** Diarrhea score and colonic glutamate concentration of healthy piglets treated with diets supplemented with glutamate. **g** Diarrhea score of healthy piglets treated with glutamate through rectal administration. n=6, Data are represented as mean ± SEM and determined by two-tailed Student’s t-test.


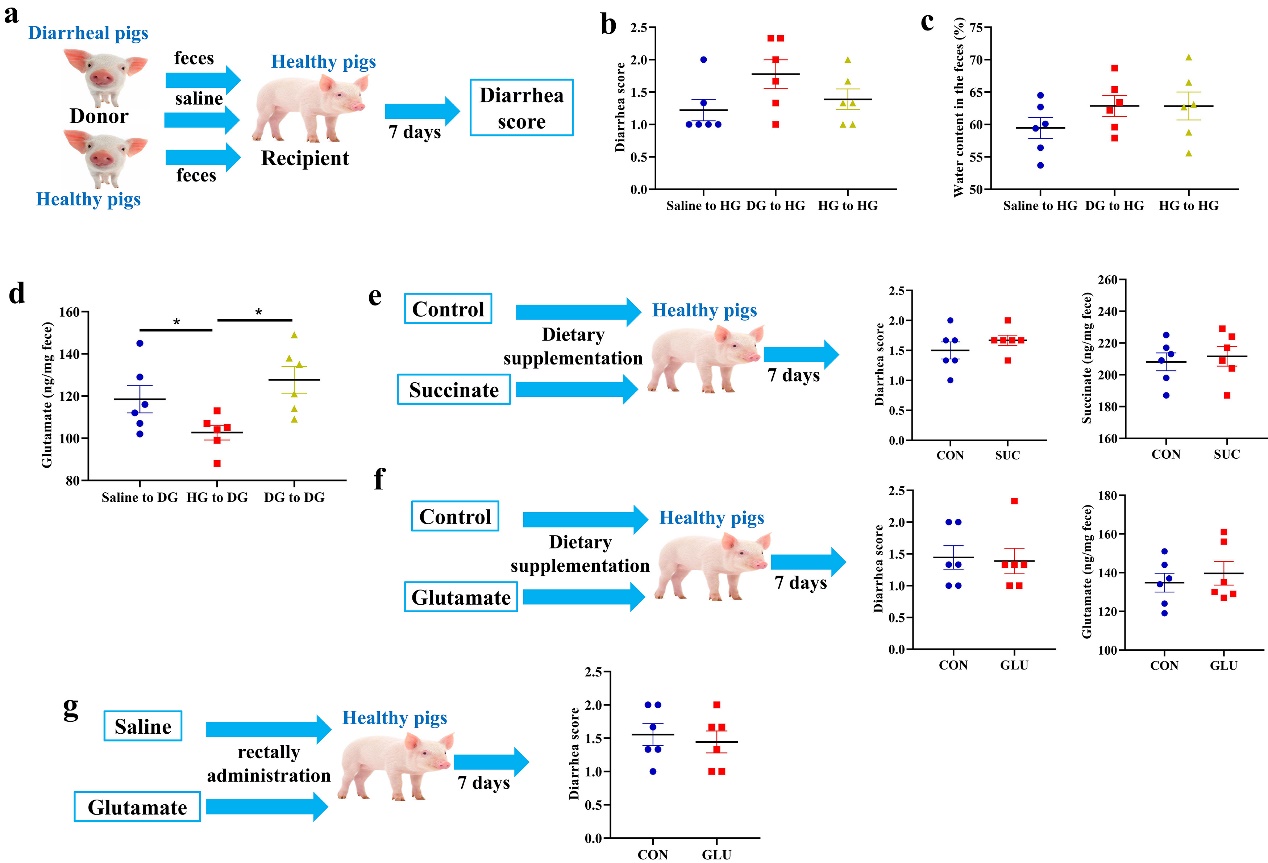


**Supplementary Fig.6 Exosome-sized extracellular vesicles in the feces of diarrheal and healthy piglets.** Scale bar, 100 nm. HEA, feces from healthy piglets; DIA, feces from diarrheal piglets. Arrows, extracellular vesicles.


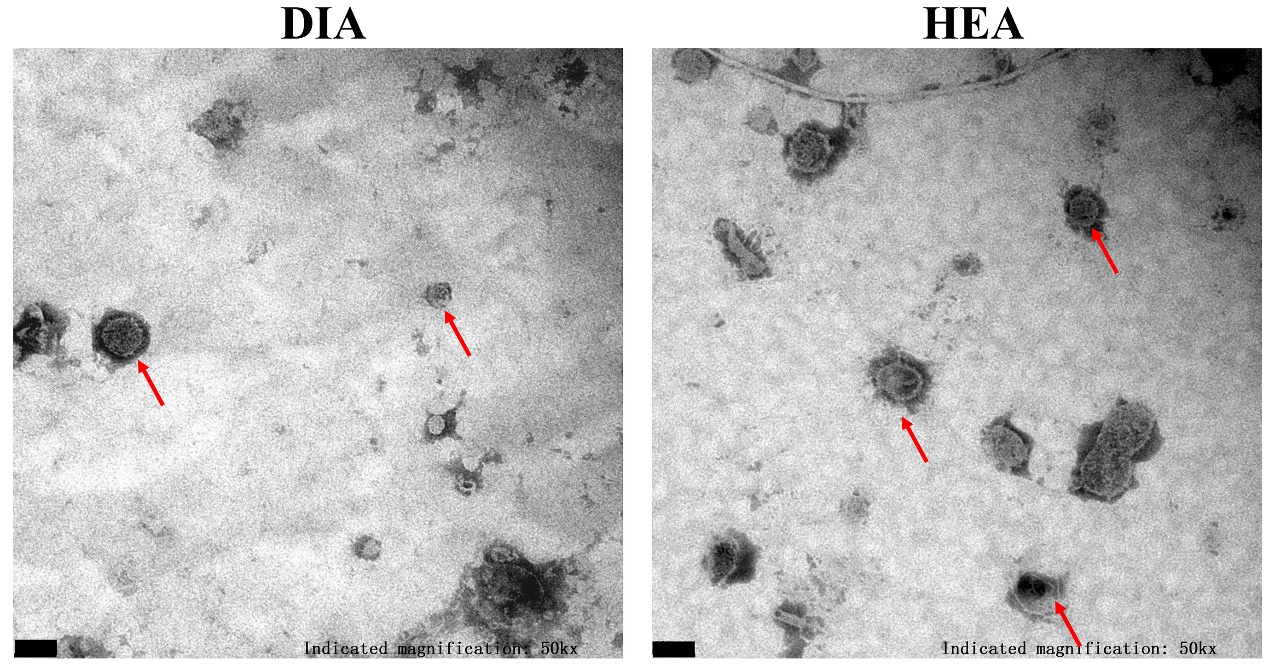


**Supplementary Fig.7 Schematic diagram of the putative binding sites of the seed sequence of ssc-miRNA-425-5p and ssc-miRNA-423-3p in the two genes (*frdi* and *frdf*) of *Prevotella*.**


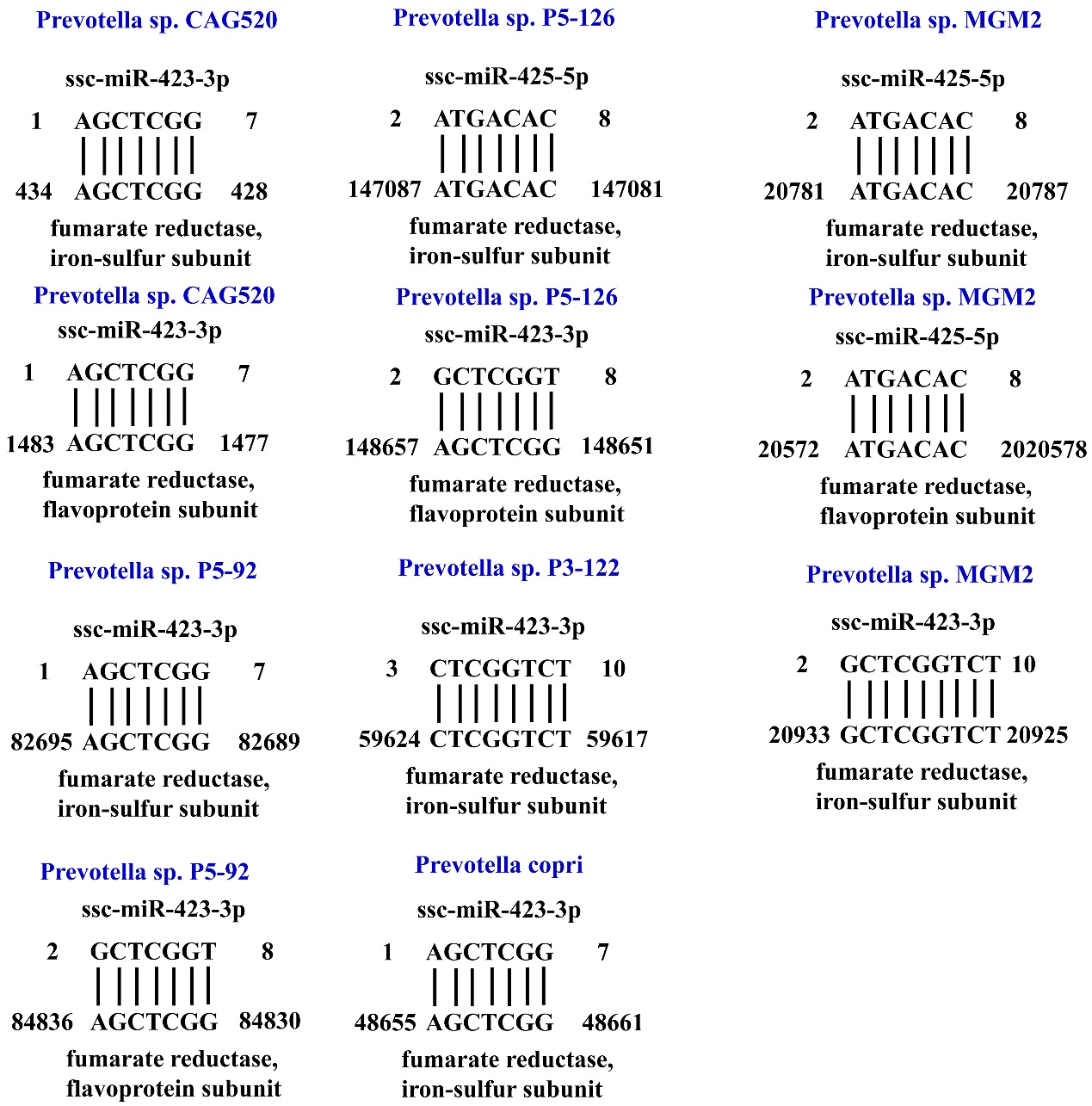


**Supplementary Fig.8 M^6^A methylation regulated the splicing process of pri-ssc-miRNA-425-5p and pri-ssc-miRNA-423-3p. a** Ssc-miRNA-425-5p and pri-ssc-miRNA-425-5p expression in the colonic tissue (n=6). **b** Ssc-miRNA-423-3p and pri-ssc-miRNA-423-3p expression in the colonic tissue (n=6). **c, d** M^6^A modiﬁed pri-ssc-miRNA-425-5p (**c**) and pri-ssc-miRNA-423-3p (**d**) levels were detected by m^6^A antibody (n=3). Data are represented as mean ± SEM. **P*<0.05, ***P*<0.01, determined by two-tailed Student’s t-test. ns, not significant. HEA, healthy piglets without diarrhea; DIA, diarrheal piglets.


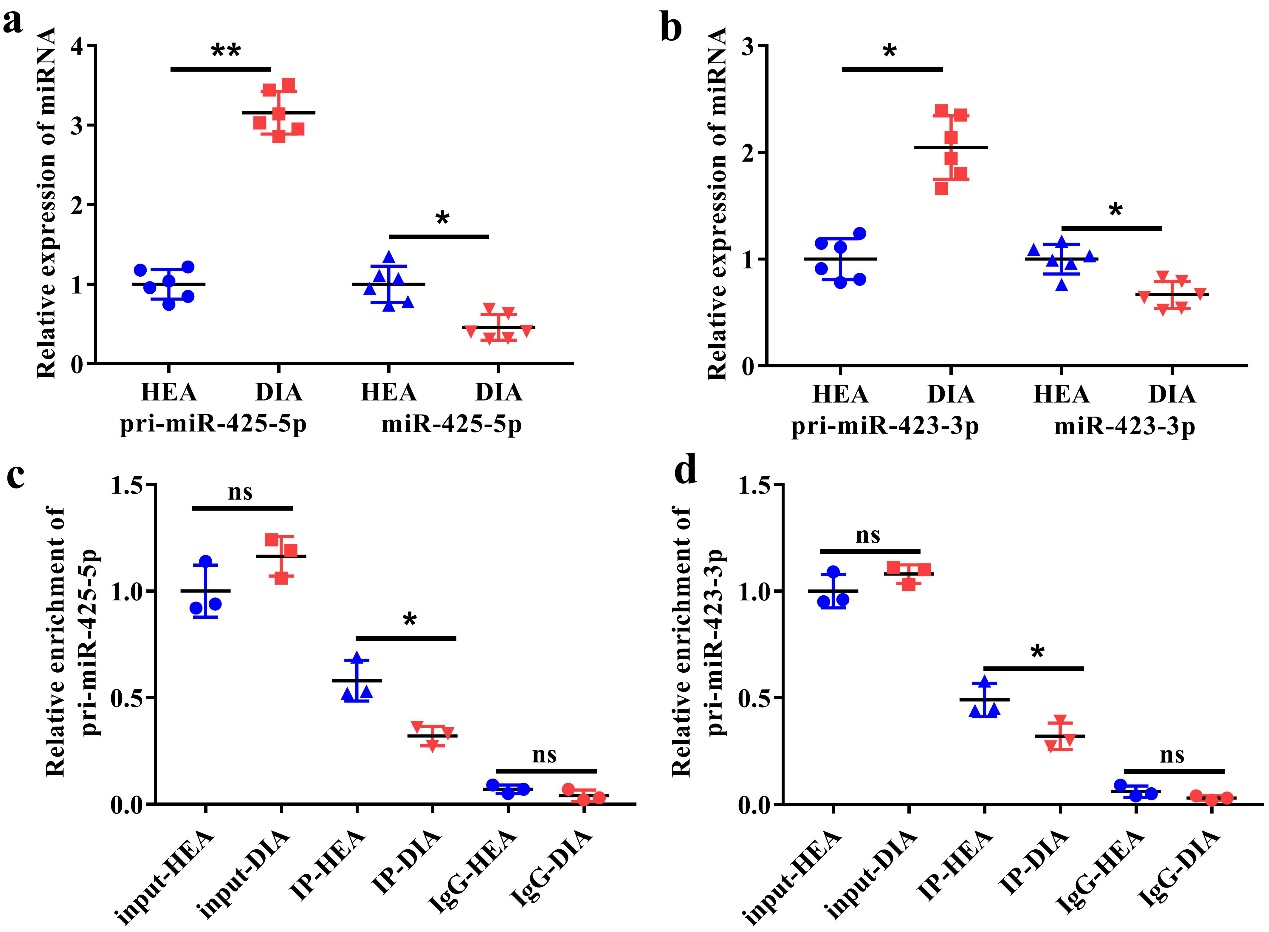


**Supplementary Fig.9 The t-SNE plot of scRNA-seq data of colonic tissue from diarrheal piglet and healthy piglet showing clusters**. **a**, merged result. **b-c**, separated results.


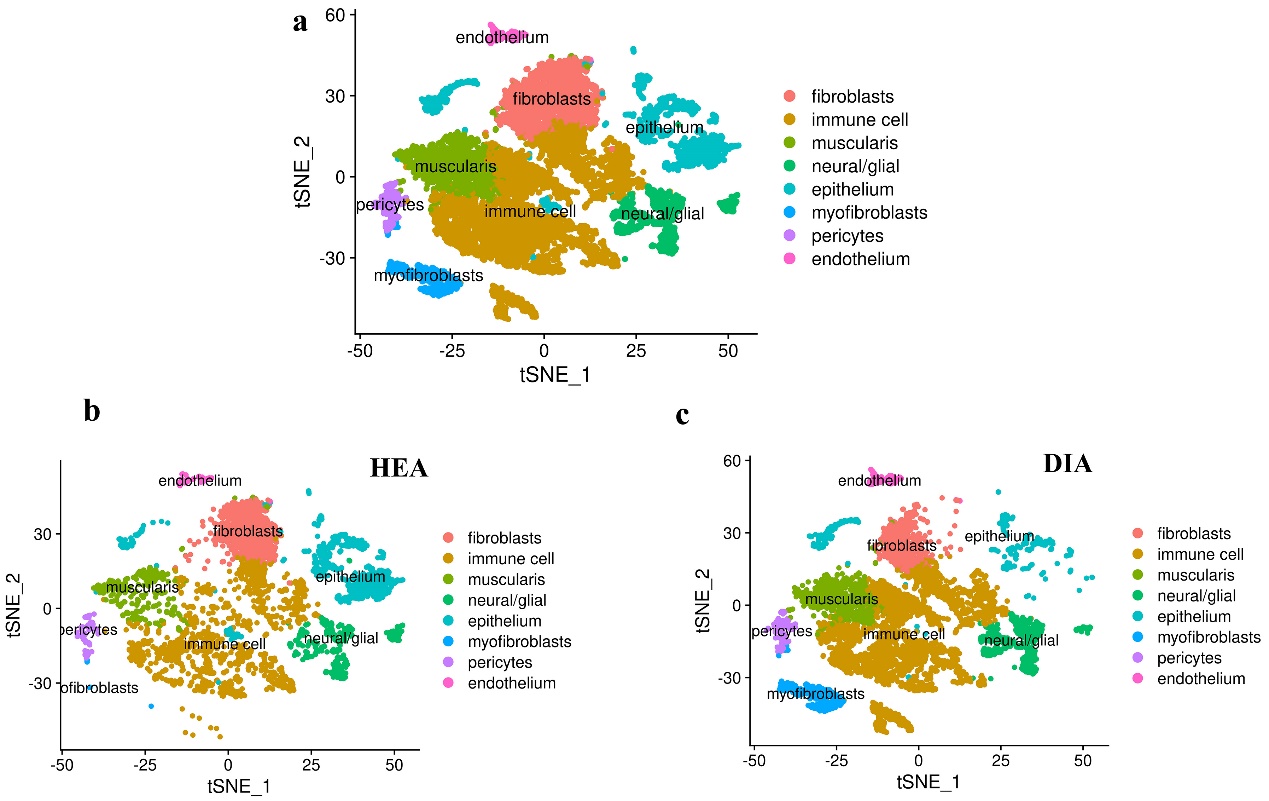


**Supplementary Fig.10 The statistics of GO enrichment of scRNA-seq data**. **a**, epithelial cells. **b**, immune cells.


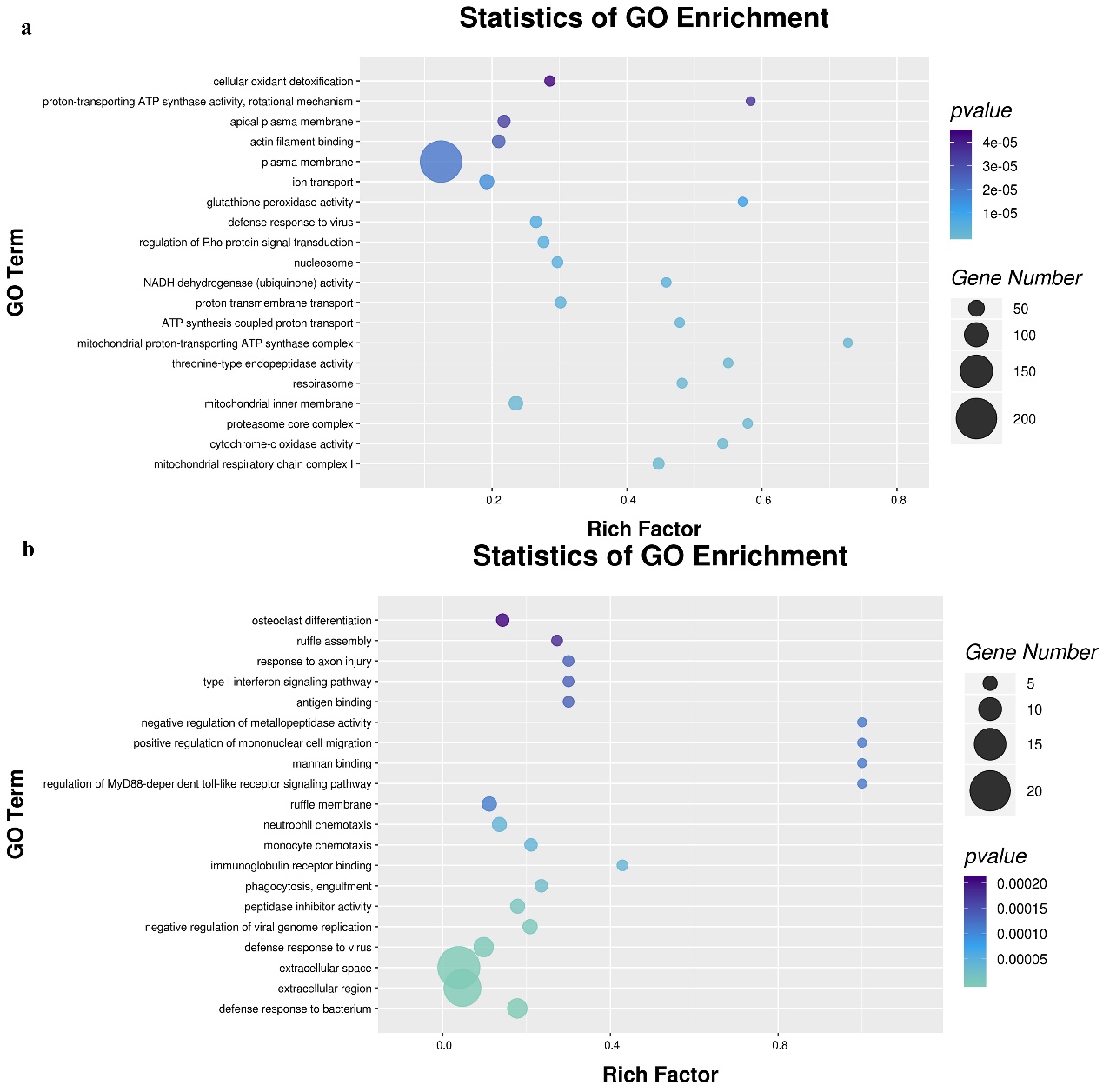


**Supplementary Fig.11 Effects of miRNA-425-5p and miRNA-423-3p mimics on gene expression in IPEC-J2 and RAW264.7 cells. a, b** Expression levels of *ANO9* (**a**) and *CLCA1* (**b**) genes in IPEC-J2 cells treated with miRNA-425-5p mimics; **c, d** Expression levels of *ANO9* (**c**) and *CLCA1* (**d**) genes in IPEC-J2 cells treated with miRNA-423-3p mimics; **e, f** Expression levels of *IRF1* (**e**) and *IRF7* (**f**) genes in RAW264.7 cells treated with miRNA-425-5p mimics; **g, h** Expression levels of *IRF1* (**g**) and *IRF7* (**h**) genes in RAW264.7 cells treated with miRNA-425-5p mimics. n=6, Data are represented as mean ± SEM and determined by two-tailed Student’s t-test.


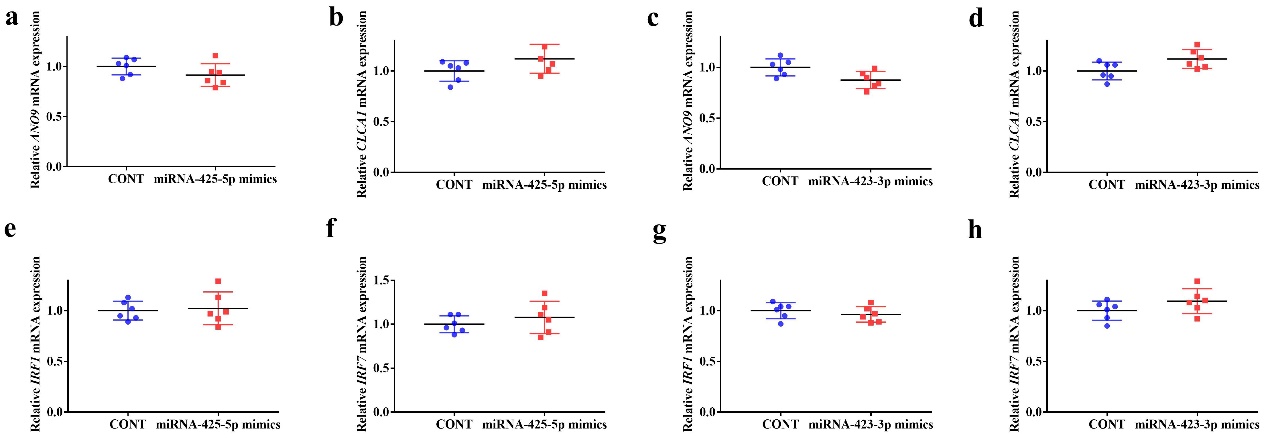

Supplement: Supplemental Material [file KGMI_A_2091369_SM1918.docx]
